# The genome sequence of the box-headed blood bee, *Sphecodes monilicornis *(Kirby, 1802)

**DOI:** 10.12688/wellcomeopenres.17786.1

**Published:** 2022-03-29

**Authors:** Steven Falk, University of Oxford and Wytham Woods Genome Acquisition Lab, Darwin Tree of Life Barcoding collective, Wellcome Sanger Institute Tree of Life programme, Wellcome Sanger Institute Scientific Operations: DNA Pipelines collective, Tree of Life Core Informatics collective, Joseph Monks, Darwin Tree of Life Consortium

**Affiliations:** 1 Independent Researcher, Kenilworth, Warwickshire, UK; 2 Department of Life Sciences, Natural History Museum, London, UK

**Keywords:** Sphecodes monilicornis, box-headed blood bee, genome sequence, chromosomal, Hymenoptera

## Abstract

We present a genome assembly from an individual male *Sphecodes monilicornis *(the box-headed blood bee; Arthropoda; Insecta; Hymenoptera; Halictidae). The genome sequence is 497 megabases in span. The majority of the assembly (95.04%) is scaffolded into 19 chromosomal pseudomolecules. The mitochondrial genome was also assembled and is 15.6 kilobases in length.

## Species taxonomy

Eukaryota; Metazoa; Ecdysozoa; Arthropoda; Hexapoda; Insecta; Pterygota; Neoptera; Endopterygota; Hymenoptera; Apocrita; Aculeata; Apoidea; Halictidae; Halictinae; Sphecodini; Sphecodes; *Sphecodes monilicornis* (Kirby, 1802) (NCBI:txid1190790).

## Background

*Sphecodes monilicornis* (Box-headed Blood Bee) is a cleptoparasitic species believed to show adult, closed-cell parasitism ( Danforth *et al*., 2019). Active between March and September, females enter a nest already fully provisioned by the host species. An *S. monilicornis* female reopens a cell, destroys the host’s egg, and lays her own egg. Once the larvae hatch, they feed on the pollen supply left by the original host. While individual females tend to specialise on one host species, the species as a whole is a host generalist, parasitising several different genera ( Bogusch *et al.*, 2006). In the UK this includes *Halictus rubicundus* (Christ), *Lasioglossum malachurum* (Kirby), *L. albipes* (Fabricius), *L. calceatum* (Scopoli), *L. laticeps* (Schenck), *L. xanthopus* (Kirby) and *L. zonulum* (Smith). Widespread in southern England the species abundance declines northwards. Outside of the UK the range of *S. monilicornis* extends to the far east of Russia and as far south as North Africa and Pakistan. As a cleptoparasite females do not provision nests themselves but have been recorded visiting composites and umbellifers ( Falk, 2015).

## Genome sequence report

**Figure 1.**
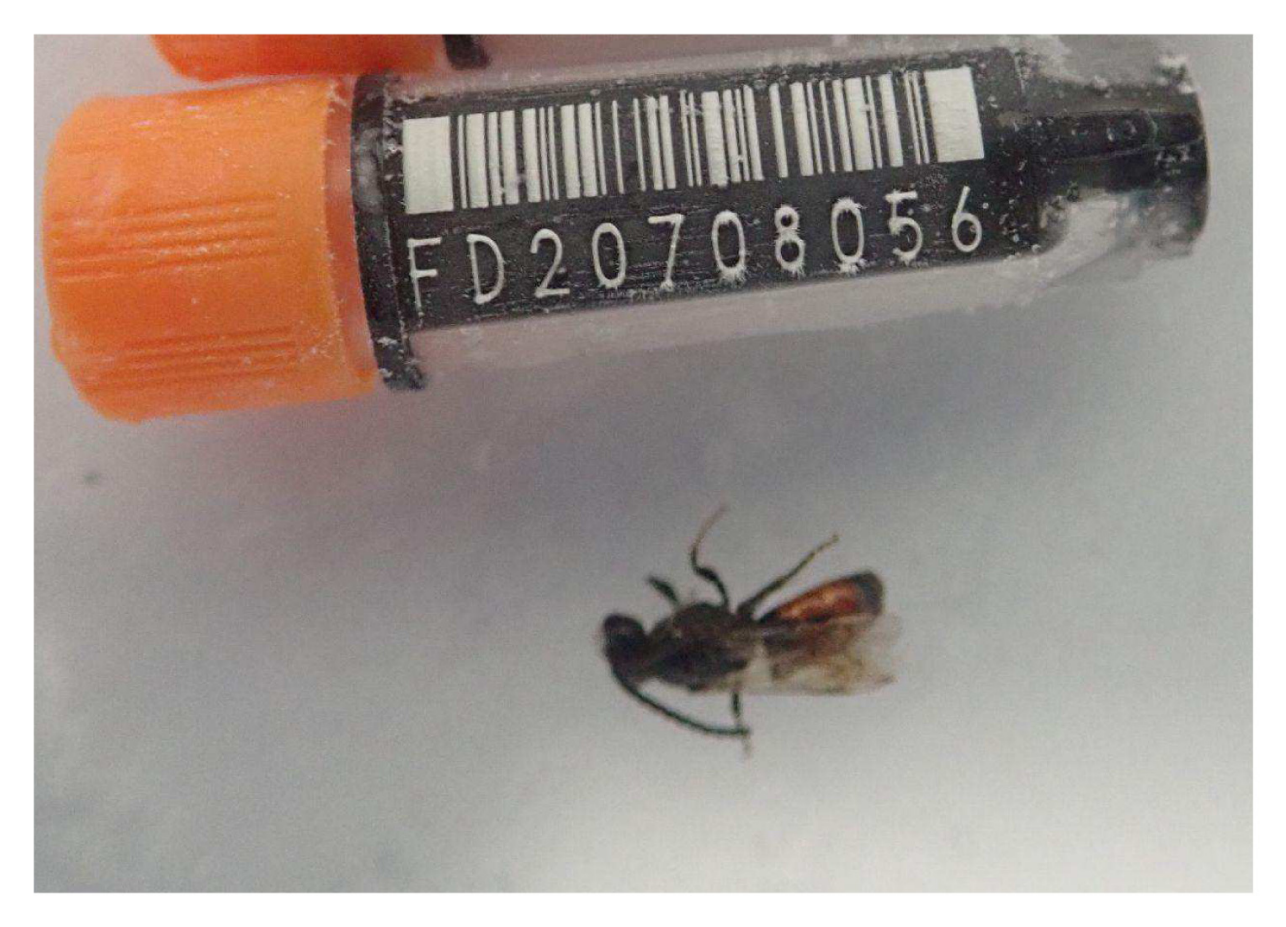
Image of the *Sphecodes monilicornis* (iySphMoni1) specimen taken during preservation and processing.

The genome was sequenced from a single male *S. monilicornis* ( Figure 1) collected from Wytham Woods, Oxfordshire (biological vice-county: Berkshire), UK (latitude 51.769, longitude -1.339). A total of 34-fold coverage in Pacific Biosciences single-molecule long reads and 80-fold coverage in 10X Genomics read clouds were generated. Primary assembly contigs were scaffolded with chromosome conformation Hi-C data. Manual assembly curation corrected 45 missing/misjoins, increasing the assembly size by 0.97%, the scaffold number by 29.12% and the scaffold N50 by 16.25%.

**Figure 2.**
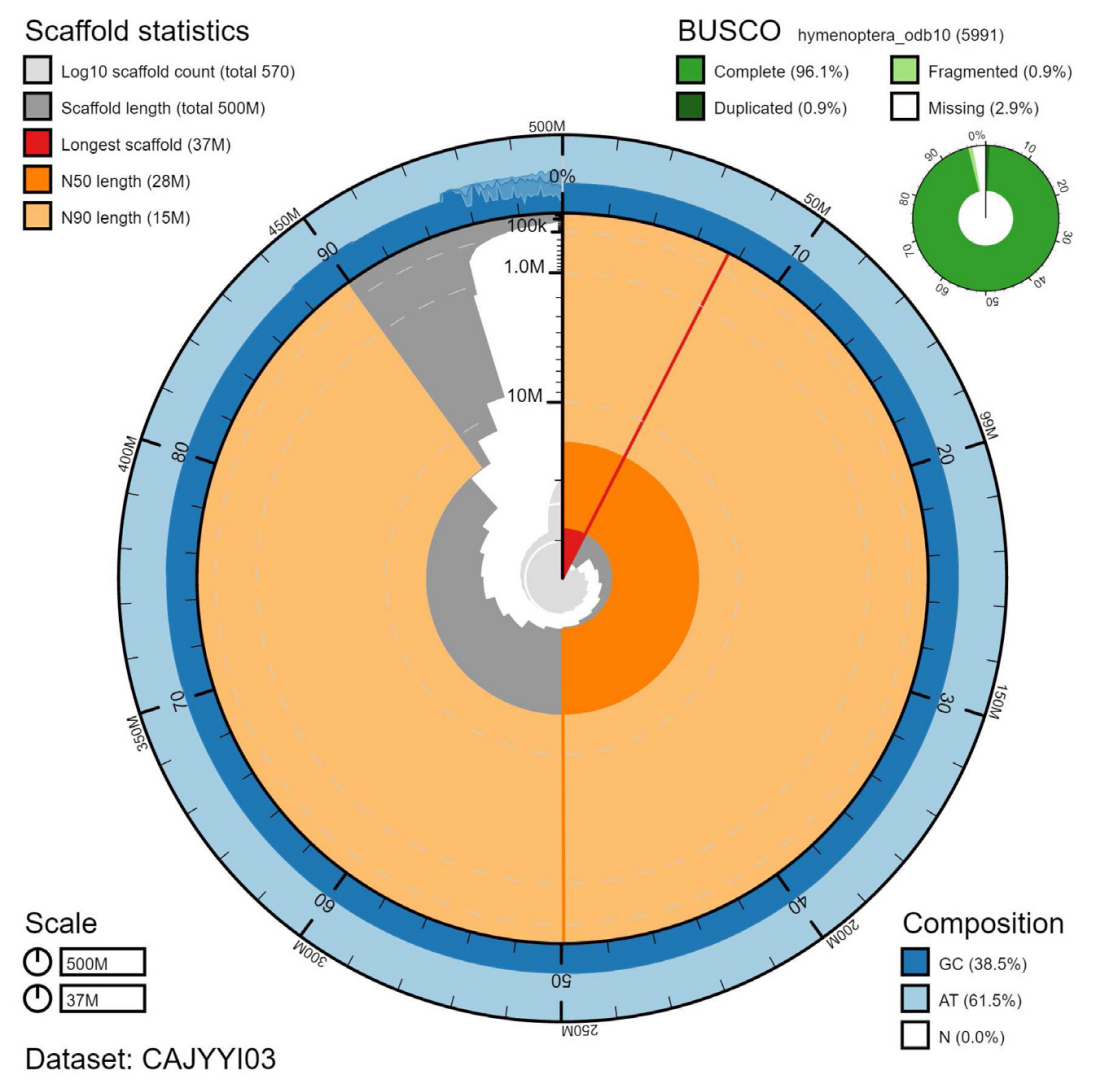
Genome assembly of *Sphecodes monilicornis*, iySphMoni1.3: metrics. The BlobToolKit Snailplot shows N50 metrics and BUSCO gene completeness. The main plot is divided into 1,000 size-ordered bins around the circumference with each bin representing 0.1% of the 496,587,026 bp assembly. The distribution of scaffold lengths is shown in dark grey with the plot radius scaled to the longest scaffold present in the assembly (37,309,700 bp, shown in red). Orange and pale-orange arcs show the N50 and N90 scaffold lengths (27,521,247 and 14,654,763 bp), respectively. The pale grey spiral shows the cumulative scaffold count on a log scale with white scale lines showing successive orders of magnitude. The blue and pale-blue area around the outside of the plot shows the distribution of GC, AT and N percentages in the same bins as the inner plot. A summary of complete, fragmented, duplicated and missing BUSCO genes in the hymenoptera_odb10 set is shown in the top right. An interactive version of this figure is available at https://blobtoolkit.genomehubs.org/view/iySphMoni1.3/dataset/CAJYYI03/snail.

The final assembly has a total length of 497 Mb in 572 sequence scaffolds with a scaffold N50 of 27.5 Mb ( Table 1). Of the assembly sequence, 95.04% was assigned to 19 chromosomal-level scaffolds (numbered by sequence length) ( Figure 2– Figure 5; Table 2). The assembly has a BUSCO v5.2.2 ( Manni *et al*., 2021) completeness of 96.1% (single 95.3%, duplicated 0.9%) using the hymenoptera_odb10 reference set (n=5991).

**Table 1.** Genome data for *Sphecodes monilicornis*, iySphMoni1.3.

*Project accession data*	
Assembly identifier	iySphMoni1.3
Species	*Sphecodes monilicornis*
Specimen	iySelTumu1
NCBI taxonomy ID	NCBI:txid1190790
BioProject	PRJEB45672
BioSample ID	SAMEA7746755
Isolate information	Thorax (genome assembly); Hi-C (head)
*Raw data accessions*	
PacificBiosciences SEQUEL II	RR6939223
10X Genomics Illumina	ERR6363305-ERR6363308
Hi-C Illumina	ERR6363309
*Genome assembly*	
Assembly accession	GCA_913789915.3
Span (Mb)	497
Number of contigs	743
Contig N50 length (Mb)	7.4
Number of scaffolds	572
Scaffold N50 length (Mb)	27.5
Longest scaffold (Mb)	37.3
BUSCO * genome score	C:96.1%[S:95.3%,D:0.9%], F:0.9%,M:2.9%,n:5991

*BUSCO scores based on the hymenoptera_odb10 BUSCO set using v5.2.2. C= complete [S= single copy, D=duplicated], F=fragmented, M=missing, n=number of orthologues in comparison. A full set of BUSCO scores is available at https://blobtoolkit.genomehubs.org/view/iySphMoni1.3/dataset/CAJYYI03/busco.

**Figure 3.**
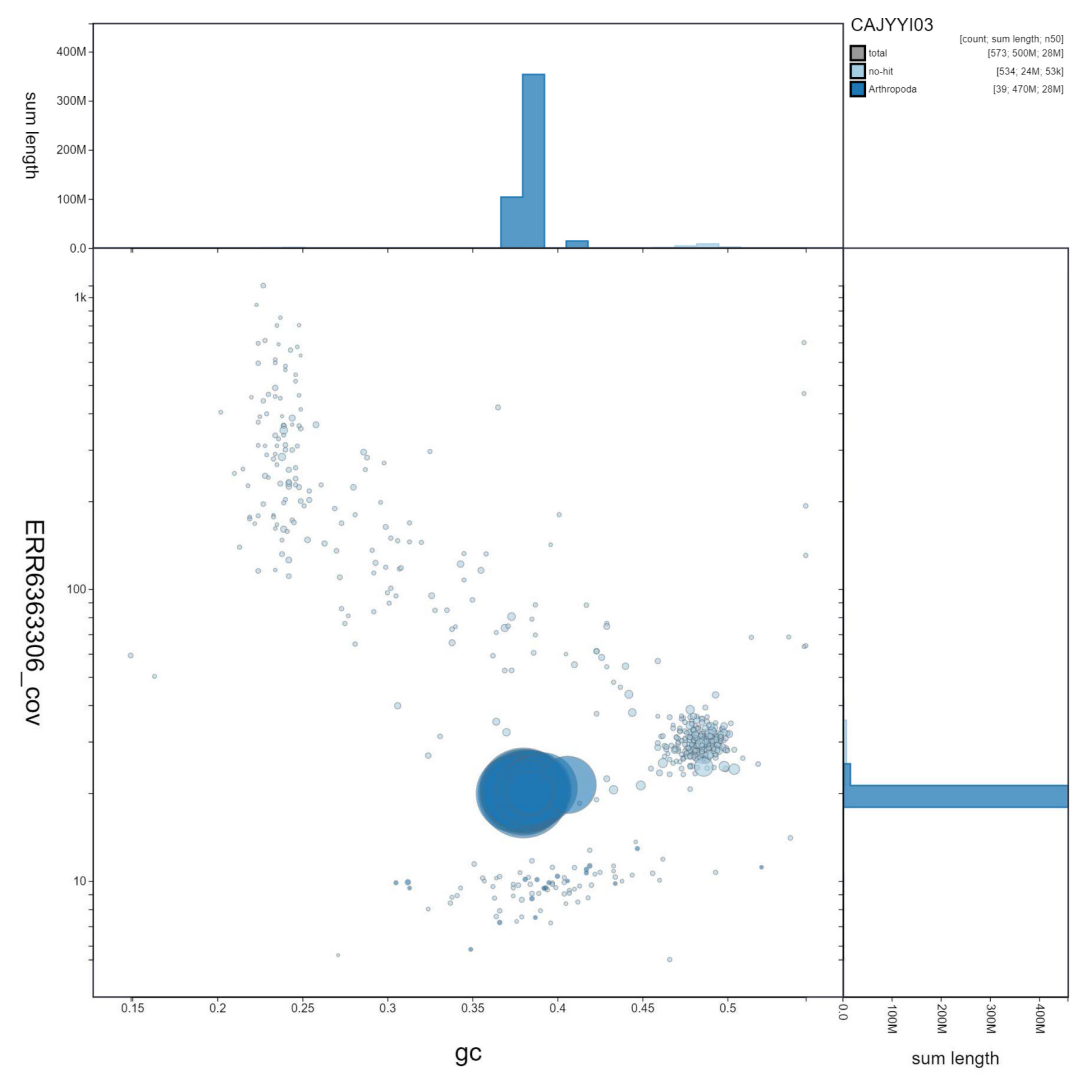
Genome assembly of *Sphecodes monilicornis*, iySphMoni1.3. GC coverage. BlobToolKit GC-coverage plot. Scaffolds are coloured by phylum. Circles are sized in proportion to scaffold length. Histograms show the distribution of scaffold length sum along each axis. An interactive version of this figure is available at https://blobtoolkit.genomehubs.org/view/iySphMoni1.3/dataset/CAJYYI03/blob.

**Figure 4.**
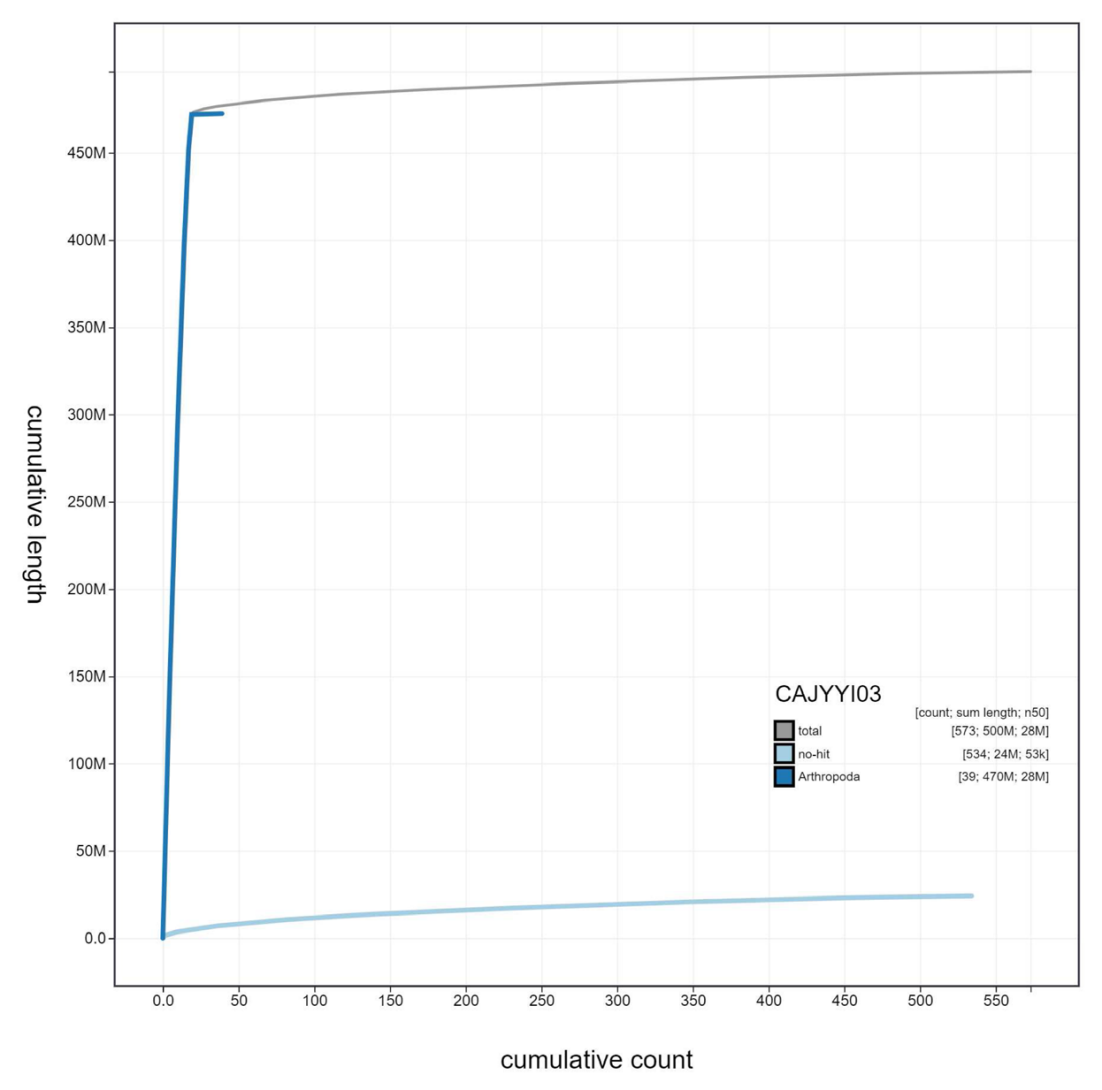
Genome assembly of *Sphecodes monilicornis*, iySphMoni1.3: cumulative sequence. BlobToolKit cumulative sequence plot. The grey line shows cumulative length for all scaffolds. Coloured lines show cumulative lengths of scaffolds assigned to each phylum using the buscogenes taxrule. An interactive version of this figure is available at https://blobtoolkit.genomehubs.org/view/iySphMoni1.3/dataset/CAJYYI03/cumulative.

**Figure 5.**
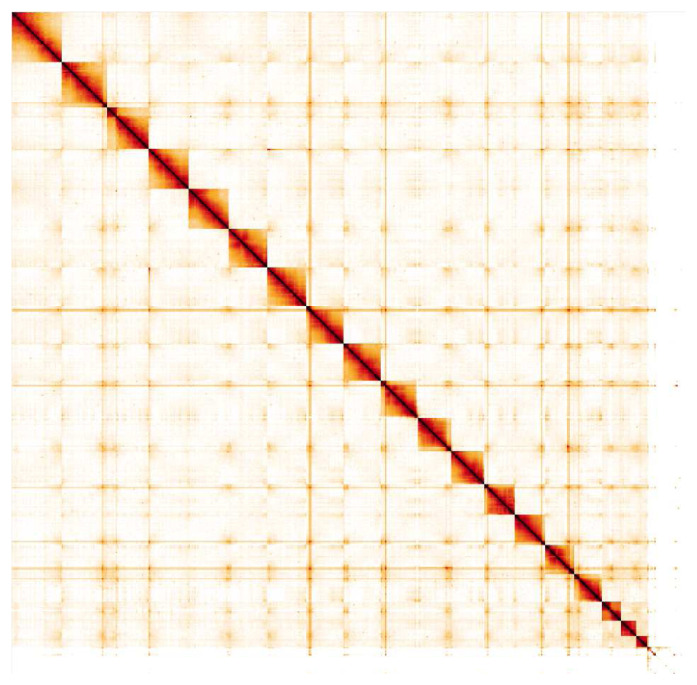
Genome assembly of *Sphecodes monilicornis*, iySphMoni1.3: Hi-C contact map. Hi-C contact map of the iySphMoni1.3 assembly, visualised in HiGlass. Chromosomes are shown in size order from left to right and top to bottom. An interactive version of this map is available here.

**Table 2.** Chromosomal pseudomolecules in the genome assembly of *Sphecodes monilicornis*, iySphMoni1.3.

INSDC accession	Chromosome	Size (Mb)	GC%
OU565284.1	1	37.31	38.0
OU565285.1	2	33.82	38.0
OU565286.1	3	30.69	38.1
OU565287.1	4	30.12	38.3
OU565288.1	5	29.46	38.1
OU565289.1	6	28.86	38.0
OU565290.1	7	28.49	38.5
OU565291.1	8	27.86	38.2
OU565292.1	9	27.52	37.5
OU565293.1	10	26.91	37.7
OU565294.1	11	25.19	37.9
OU565295.1	12	24.20	37.7
OU565296.1	13	22.73	38.5
OU565297.1	14	22.34	39.1
OU565298.1	15	21.54	38.7
OU565299.1	16	20.22	38.3
OU565300.1	17	14.65	40.6
OU565301.1	18	11.56	38.4
OU565302.1	19	8.47	38.4
OU565303.1	MT	0.02	16.4
-	Unplaced	24.62	43.4

## Methods

### Sample acquisition and DNA extraction

A male *S. monilicornis* specimen (iySphMoni1) was collected from Wytham Woods, Oxfordshire (biological vice-county: Berkshire), UK (latitude 51.769, longitude -1.339) by Steven Falk, Independent Researcher, from woodland using a net. The specimen was identified by the same individual and snap-frozen on dry ice.

DNA was extracted at the Tree of Life laboratory, Wellcome Sanger Institute. The iySelTumu1 sample was weighed and dissected on dry ice. Thorax tissue was disrupted using a Nippi Powermasher fitted with a BioMasher pestle. Fragment size analysis of 0.01–0.5 ng of DNA was then performed using an Agilent FemtoPulse. High molecular weight (HMW) DNA was extracted using the Qiagen MagAttract HMW DNA extraction kit. Low molecular weight DNA was removed from a 200-ng aliquot of extracted DNA using 0.8X AMpure XP purification kit prior to 10X Chromium sequencing; a minimum of 50 ng DNA was submitted for 10X sequencing. HMW DNA was sheared into an average fragment size between 12-20 kb in a Megaruptor 3 system with speed setting 30. Sheared DNA was purified by solid-phase reversible immobilisation using AMPure PB beads with a 1.8X ratio of beads to sample to remove the shorter fragments and concentrate the DNA sample. The concentration of the sheared and purified DNA was assessed using a Nanodrop spectrophotometer and Qubit Fluorometer and Qubit dsDNA High Sensitivity Assay kit. Fragment size distribution was evaluated by running the sample on the FemtoPulse system.

### Sequencing

Pacific Biosciences HiFi circular consensus and 10X Genomics read cloud sequencing libraries were constructed according to the manufacturers’ instructions. Sequencing was performed by the Scientific Operations core at the Wellcome Sanger Institute on Pacific Biosciences SEQUEL II and Illumina NovaSeq 6000 instruments. Hi-C data were generated from head tissue of iySphMoni1 using the Arima v2.0 kit and sequenced on an Illumina NovaSeq 6000 instrument.

### Genome assembly

Assembly was carried out with Hifiasm ( Cheng *et al*., 2021). Haplotypic duplication was identified and removed with purge_dups ( Guan *et al*., 2020). Scaffolding with Hi-C data ( Rao *et al*., 2014) was carried out with SALSA2 ( Ghurye *et al*., 2019). The Hi-C scaffolded assembly was polished with the 10X Genomics Illumina data by aligning to the assembly with longranger align, calling variants with freebayes ( Garrison & Marth, 2012). One round of the Illumina polishing was applied. The mitochondrial genome was assembled with MitoHiFi ( Uliano-Silva *et al*., 2021), which performed annotation using MitoFinder ( Allio *et al*., 2020). The assembly was checked for contamination as described previously ( Howe *et al*., 2021). Manual curation ( Howe *et al*., 2021) was performed using HiGlass ( Kerpedjiev *et al*., 2018) and Pretext. The genome was analysed within the BlobToolKit environment ( Challis *et al*., 2020). Table 3 contains a list of all software tool versions used, where appropriate.

**Table 3.** Software tools used.

Software tool	Version	Source
Hifiasm	0.14	Cheng *et al*., 2021
purge_dups	1.2.3	Guan *et al*., 2020
SALSA2	2.2	Ghurye *et al*., 2019
longranger align	2.2.2	https://support.10xgenomics. com/genome-exome/software/ pipelines/latest/advanced/other- pipelines
freebayes	v1.3.1-17-gaa2ace8	Garrison & Marth, 2012
MitoHiFi	2	Uliano-Silva *et al*., 2021
HiGlass	1.11.6	Kerpedjiev *et al*., 2018
PretextView	0.2.x	https://github.com/wtsi-hpag/PretextView
BlobToolKit	3.0.5	Challis *et al*., 2020

### Ethics/compliance issues

The materials that have contributed to this genome note have been supplied by a Darwin Tree of Life Partner. The submission of materials by a Darwin Tree of Life Partner is subject to the Darwin Tree of Life Project Sampling Code of Practice. By agreeing with and signing up to the Sampling Code of Practice, the Darwin Tree of Life Partner agrees they will meet the legal and ethical requirements and standards set out within this document in respect of all samples acquired for, and supplied to, the Darwin Tree of Life Project. Each transfer of samples is further undertaken according to a Research Collaboration Agreement or Material Transfer Agreement entered into by the Darwin Tree of Life Partner, Genome Research Limited (operating as the Wellcome Sanger Institute), and in some circumstances other Darwin Tree of Life collaborators.

## Data availability

European Nucleotide Archive: Sphecodes monilicornis (box-headed blood bee). Accession number PRJEB45672; https://identifiers.org/ena.embl/PRJEB45672.

The genome sequence is released openly for reuse. The *S. monilicornis* genome sequencing initiative is part of the Darwin Tree of Life (DToL) project. All raw sequence data and the assembly have been deposited in INSDC databases. The genome will be annotated and presented through the Ensembl pipeline at the European Bioinformatics Institute. Raw data and assembly accession identifiers are reported in Table 1.

## Author information

Members of the University of Oxford and Wytham Woods Genome Acquisition Lab are listed here: https://doi.org/10.5281/zenodo.5746938.

Members of the Darwin Tree of Life Barcoding collective are listed here: https://doi.org/10.5281/zenodo.5744972.

Members of the Wellcome Sanger Institute Tree of Life programme are listed here: https://doi.org/10.5281/zenodo.6125027.

Members of Wellcome Sanger Institute Scientific Operations: DNA Pipelines collective are listed here: https://doi.org/10.5281/zenodo.5746904.

Members of the Tree of Life Core Informatics collective are listed here: https://doi.org/10.5281/zenodo.6125046.

Members of the Darwin Tree of Life Consortium are listed here: https://doi.org/10.5281/zenodo.5638618.
