# Supplementary material for: The genome sequence of the box-headed blood bee, Sphecodes monilicornis (Kirby, 1802)
Source: Wellcome Open Res. 2022 Mar 29;7:115. [Version 1] doi: 10.12688/wellcomeopenres.17786.1 (PMC10034205; doi:10.12688/wellcomeopenres.17786.1)
Supplement: Peer review history

# Peer review: Reviewer response for version 1

**DOI:** 10.21956/wellcomeopenres.19685.r55477

**Published:** 2023-03-22

**Authors:** Gabriel Luz Wallau

**Affiliations:** 1 Instituto Aggeu Magalhães (IAM), Fundação Oswaldo Cruz (FIOCRUZ/PE),, Recife, Pernambuco, Brazil

This data note manuscript reports the genome sequencing of a cleptoparasitic bee. The results are presented in a compact and direct format with all the relevant information. The authors obtained a high quality genome assembly employing complementary sequencing and bioinformatic methods. Moreover, the data availability and repository links have been provided were I could check that the data is available with enough details regarding access and reproducibility. The only part I think should be improved is the quality check and filtering of low quality reads. I could not find this information in the Methods section. I suggest to add it between Sequencing and Genome Assembly subheadings as a new subsection.

Are sufficient details of methods and materials provided to allow replication by others?

Partly

Is the rationale for creating the dataset(s) clearly described?

Yes

Are the datasets clearly presented in a useable and accessible format?

Yes

Are the protocols appropriate and is the work technically sound?

Yes

Reviewer Expertise:

Genomics of pathogens and vectors.

I confirm that I have read this submission and believe that I have an appropriate level of expertise to confirm that it is of an acceptable scientific standard.

# Peer review: Reviewer response for version 1

**DOI:** 10.21956/wellcomeopenres.19685.r54865

**Published:** 2023-03-08

**Authors:** Pilar De la Rúa

**Affiliations:** 1 University of Murcia,, Murcia, Spain

This manuscript presents whole genome sequencing data from a bee with interesting kleptoparasitic behaviour in that it is parasitic on other important pollinator bees.

 The paper is concise with regard to the details provided on methodology and analyses. Overall, the data adds to the growing insect genome database and it is hoped that their annotation will result in the identification of genes of interest.

 Only one small drawback, the image of the bee is not in focus.

Are sufficient details of methods and materials provided to allow replication by others?

Yes

Is the rationale for creating the dataset(s) clearly described?

Yes

Are the datasets clearly presented in a useable and accessible format?

Yes

Are the protocols appropriate and is the work technically sound?

Yes

Reviewer Expertise:

pollinators conservation genetics and molecular ecology

I confirm that I have read this submission and believe that I have an appropriate level of expertise to confirm that it is of an acceptable scientific standard.
